# Molecular identification of fluoroquinolone resistance in *Salmonella* spp. isolated from broiler farms and human samples obtained from two regions in Colombia

**DOI:** 10.14202/vetworld.2021.1767-1773

**Published:** 2021-07-07

**Authors:** María Paula Herrera-Sánchez, Rafael Enrique Castro-Vargas, Luz Clemencia Fandiño-de-Rubio, Roy Rodríguez-Hernández, Iang Schroniltgen Rondón-Barragán

**Affiliations:** 1 Research Group in Immunobiology and Pathogenesis, Laboratory of Immunology and Molecular Biology, Faculty of Veterinary Medicine and Zootechnics, University of Tolima, Santa Helena Highs, Ibagué 730006299, Tolima, Colombia; 2 Poultry Research Group, Laboratory of Immunology and Molecular Biology, Faculty of Veterinary Medicine and Zootechnics, University of Tolima, Santa Helena Highs, Ibagué 730006299, Tolima, Colombia

**Keywords:** antibiotic resistance, broiler, resistance genes, *Salmonella*

## Abstract

**Background and Aim:**

*Salmonella* is one of the most common foodborne pathogens, the emergence of antibiotic-resistant strains of which is increasing. The aim of this study was to phenotypically and genotypically characterize the fluoroquinolone resistance of *Salmonella* isolates from broiler and humans in two regions of Colombia.

**Materials and Methods:**

*Salmonella* strains (n*=*49) were evaluated. The phenotype of antibiotic resistance was assessed by an automated method and agar diffusion method, as well as the presence of the quinolone resistance genes *qnrA*, *qnrB*, *qnrC*, *qnrD*, *qnrS*, and *aac(6’)-Ib* as determined by polymerase chain reaction.

**Results:**

Strains were resistant to ciprofloxacin (75%), levofloxacin (57.1%), and enrofloxacin (38.8%). Molecular identification showed that 24 out of 49 strains possessed the *qnrB* gene (48.9%), while only one isolate from the Santander region possessed the *aac(6’)-Ib* gene. Regarding Class 1 integron, it was present in 11 out of the 49 strains (22.44%).

**Conclusion:**

This study reports the presence of the gene *qnrB* as well the presence of Class 1 integrons in broiler *Salmonella* isolates, which may contribute to the resistance to fluoroquinolones.

## Introduction

*Salmonella* spp. is one of the most common foodborne pathogens globally and has a major impact on public health [1,2]. *Salmonella enterica* comprises a diverse group classified according to their antigens; some serotypes can cause major systemic infections [1]. In the case of humans, salmonellosis can induce different clinical conditions varying widely in their severity, such as typhoid fever, paratyphoid fever, septicemia, and gastroenteritis, especially in immunocompromised individuals such as children and the elderly [3]. In terms of global numbers, *Salmonella* spp. is responsible for 93.8 million human infections and 155,000 deaths annually [4]. In Colombia between 1997 and 2017, the National Institute of Health reported *Salmonell*a spp. strains isolated from 12,055 human samples [5]. *Salmonella* is frequently transmitted to humans by the consumption of food of animal origin, especially avian products such as eggs and chicken meat [2,6]. As an aggravating factor, most clinical isolates have been reported to be multidrug-resistant, associated with the inappropriate use of antibiotics such as enrofloxacin as growth promoters in animal production [7,8].

At present, quinolones and fluoroquinolones are prescribed as the first therapeutic option for patients with acute bacterial diarrhea, but *Salmonella* has been increasingly developing resistance to this family of antibiotics [2,3]. The genetic basis of resistance to fluoroquinolones and quinolones in *Salmonella* spp. is mediated mainly by mutations in DNA gyrase and topoisomerase IV, which are the target structures of these antibiotics. In addition, modifications in the permeability of the external membrane, frequently transmitted by plasmids (plasmid-mediated quinolone resistance [PMQR]), and efflux bombs can confer resistance; this indicates that several biochemical mechanisms are involved in the resistance to quinolones and fluoroquinolones [9].

Our research group has reported the antibiotic resistance patterns of *Salmonella* spp. in poultry production as well as in other sectors in Colombia, as characterized by different genotyping methods [10-15]. However, these studies did not address the molecular mechanisms associated with resistance to quinolones and fluoroquinolones. The present study aims to characterize the phenotype behind resistance and the resistance genes *qnrA*, *qnrB*, *qnrC*, *qnrD*, *qnrS*, and *aac(6’)-Ib*, which mediate antimicrobial resistance to fluoroquinolones, in *Salmonella* isolates from poultry and humans from the Colombian regions of Tolima and Santander.

## Materials and Methods

### Ethical approval

No ethical approval was required for this study because *Salmonella* spp. strains were from the Bacterial Strain Collection of the Laboratory of Immunology and Molecular Biology of the Universidad del Tolima, which were obtained from previous research projects made by the Poultry Research Group of the Universidad del Tolima and they were approved by Bioethics Committee of the Central Office of Research from Universidad del Tolima and complied with the guidelines for animal care and use in research and teaching [11,16].

### Study period and location

This study was carried out from August 2018 to May 2019. Microbiological culture and molecular experiments were done at Laboratory of Immunology and Molecular Biology of the University of Tolima. In addition, Antibiotic resistance assay was done at Tolima Clinic.

### *Salmonella* spp. strains

A total of 49 strains of *Salmonella* spp. isolated from broiler farms and humans were included in this study. Overall, 15 strains were serotyped as *Salmonella* Heidelberg isolated from broiler farms in Santander; 24 strains were serotyped as *Salmonella* Paratyphi B isolated from broiler farms in Tolima; while 10 were serotyped as Newport (n=1), Enteritidis (n=4), Braenderup (n=1), Uganda (n=1), Typhimurium (n=2), and Grupensis (n=1) isolated from humans with gastroenteritis in Ibagué-Tolima.

### Phenotypic resistance

The phenotypic resistance to ciprofloxacin and levofloxacin was determined using an automated MicroScan system (Beckman Coulter, Porterville, CA, USA) and BD Phoenix NMIC/ID-94 (Becton Dickinson, Franklin Lakes, NJ, USA) through the ­minimum inhibitory concentration (MIC) method following the recommendations of the CLSI [17]. The resistance to enrofloxacin (5 mg) was determined by the Kirby–Bauer disk diffusion susceptibility test. A bacterial suspension was spread in Mueller-Hinton agar (Oxoid, Wesel, Germany) according to the McFarland turbidity scale of 0.5; then, bacterial growth inhibition was evaluated at 37°C for 18 h according to the CLSI guidelines [17].

### Genomic DNA (gDNA) extraction

gDNA was extracted from fresh colonies using the Invisorb^®^ Spin Universal Kit (Stratec, Berlin, Germany); then, the samples were dissolved in 50 μL of TE buffer and maintained at −20°C until further use. In addition, all isolates were confirmed by polymerase chain reaction (PCR) through amplification of the *invA* gene using the following primers, forward 5′-TGAAATTATCGCCACGTTCGGGCAA-3′ and reverse 5′-TCATCGCACCGTCAAAGGAACC-3′, with an amplicon size of 284 bp [10]. *S*. *enterica* ATCC^®^ 13076 strain (ATCC, Manassas, VA, USA) was used as a positive control. The reaction was carried out in a total volume of 25 μL, composed of 14.87 μL of distilled-deionized water, 5 μL of 5× colorless GoTaq^®^ Flexi Buffer (Promega, Madison, WI, USA), 1 μL of dNTPs (1.5 mM) (Invitrogen, Waltham, MA, USA), 1 μL of each primer (forward and reverse) (10 pmol/μL), 1 μL of MgCl_2 (_25 mM), 0.125 μL of GoTaq^®^ Flexi DNA polymerase (Promega), and 1 μL of gDNA as a template. The amplification was carried out in a T100 thermocycler (Bio-Rad, Hercules, CA, USA) with an initial denaturation step at 95°C for 3 min, followed by 35 cycles of denaturation at 95°C for 30 s, annealing at 55°C for 30 s, extension at 72°C for 30 s, and a final extension step at 72°C for 7 min. Amplicons were revealed on 2% agarose gel by electrophoresis (PowerPac™ HC, Bio-Rad, Hercules) using the 100 bp DNA ladder Load Ready™ (Amplyus, Cambridge, MA, USA). The gel was stained with HydraGreen™ (ACTGene, Piscataway, NJ, USA) and visualized under UV light using the ENDURO™ GDS gel documentation system (Labnet International, Inc., Edison, NJ, USA).

### PMQR and Class 1 integron detection

For PMQR and Class 1 integron detection, gDNA from isolates was used as a template for the reaction, using gene-specific primer sets (Table-1) [18-22]. PCR conditions were as described above and the annealing temperature was adjusted depending on the melting temperature of each primer set.

**Table-1:** Primers used to evaluate the presence of PMQR genes in *Salmonella* spp. strains.

Target gene	Primer sequence	Annealing temperature (°C)	Amplicon size (bp)	Reference
*qnrA*	F- CCGCTTTTATCAGTGTGACT R- ACTCTATGCCAAAGCAGTTG	55	188	This study
*qnrB*	F- GATCGTGAAAGCCAGAAAGG R- ACGATGCCTGGTAGTTGTCC	54	469	[18]
*qnrC*	F- GGGTTGTACATTTATTGAATCG R- CACCTACCCATTTATTTTCA	54	308	[19]
*qnrD*	F- CGAGATCAATTTACGGGGAATA R- AACAAGCTGAAGCGCCTG	57	582	[20]
*qnrS*	F- ACGACATTCGTCAACTGCAA R- TAAATTGGCACCCTGTAGGC	55	417	[18]
*aac(6’)-Ib*	F- TTGCGATGCTCTATGAGTGGCTA R- CTCGAATGCCTGGCGTGTTT	57	482	[21]
Class 1 Integron (Integrase)	F- TCCACGCATCGTCAGGC R- CCTCCCGCACGATGATC	55	280	[22]

## Results

### Phenotypic resistance to antibiotics

Enrofloxacin resistance was present in 19/49 isolates (38.8%), while 75% of the isolates showed resistance to ciprofloxacin (36/49). In the case of levofloxacin, among 28 isolates tested for this antibiotic, 57.1% (16/28) were resistant. None of the strains isolated from humans showed resistance to these three antibiotics (Table-2).

**Table-2:** Phenotypic and genotypic characteristics of *Salmonella* spp. isolates from broiler and human samples.

Serotype	Source	*qnr*	Class 1 integron	Phenotypic resistance
*S.* Heidelberg	BS	*aac(6’)-Ib*	-	CIP, LVX
*S.* Heidelberg	BS	-	+	CIP, LVX
*S.* Heidelberg	BS	-	+	CIP, LVX
*S.* Heidelberg	BS	-	+	CIP, LVX
*S.* Heidelberg	BS	*qnrB*	-	CIP, LVX
*S.* Heidelberg	BS	-	+	CIP, LVX
*S.* Heidelberg	BS	-	-	CIP, LVX
*S.* Heidelberg	BS	-	+	CIP, LVX
*S.* Heidelberg	BS	-	+	CIP, LVX
*S.* Heidelberg	BS	-	+	CIP, LVX
*S.* Heidelberg	BS	*qnrB*	+	CIP, LVX, ENR
*S.* Heidelberg	BS	-	+	CIP, LVX
*S.* Heidelberg	BS	*qnrB*	+	CIP, LVX, ENR
*S.* Heidelberg	BS	-	+	CIP, LVX
*S.* Heidelberg	BS	-	-	CIP, LVX, ENR
*S.* Paratyphi B	BT	*qnrB*	-	CIP, ENR
*S.* Paratyphi B	BT	*qnrB*	-	CIP, LVX, ENR
*S.* Paratyphi B	BT	*qnrB*	-	CIP, ENR
*S.* Paratyphi B	BT	*qnrB*	-	ENR
*S.* Paratyphi B	BT	*qnrB*	-	CIP, ENR
*S.* Paratyphi B	BT	*qnrB*	-	CIP, ENR
*S.* Paratyphi B	BT	*qnrB*	-	ENR
*S.* Paratyphi B	BT	*qnrB*	-	CIP, ENR
*S.* Paratyphi B	BT	*qnrB*	-	CIP, ENR
*S.* Paratyphi B	BT	*qnrB*	-	CIP, ENR
*S.* Paratyphi B	BT	*qnrB*	-	CIP
*S.* Paratyphi B	BT	*qnrB*	-	CIP, ENR
*S.* Paratyphi B	BT	*qnrB*	-	-
S. Paratyphi B	BT	*qnrB*	-	CIP, ENR
*S.* Paratyphi B	BT	*qnrB*	-	CIP, ENR
*S.* Paratyphi B	BT	*qnrB*	-	CIP ENR
*S.* Paratyphi B	BT	*qnrB*	-	CIP
*S.* Paratyphi B	BT	*qnrB*	-	CIP
*S.* Paratyphi B	BT	*qnrB*	-	CIP, ENR
*S.* Paratyphi B	BT	*qnrB*	-	CIP
*S.* Paratyphi B	BT	-	-	CIP
*S.* Paratyphi B	BT	-	-	CIP
*S.* Paratyphi B	BT	*qnrB*	-	CIP, ENR
*S.* Paratyphi B	BT	-	-	CIP
*S.* Newport	H	-	-	-
*S.* Enteritidis	H	-	-	-
*S.* Enteritidis	H	-	-	-
*S.* Enteritidis	H	-	-	-
*S.* Braenderup	H	-	-	-
*S.* Uganda	H	-	-	-
*S.* Enteritidis	H	-	-	-
*S.* Typhimurium	H	-	-	-
*S.* Grupensis	H	-	-	-
*S.* Typhimurium	H	-	-	-

*qnr:* quinolone resistance gene; BS: Santander broiler chicken; BT: Tolima broiler chicken; H: Human;

CIP: ciprofloxacin; LVX: levofloxacin; ENR: enrofloxacin;

+: presence of the mobile element

### PMQR and Class 1 integron detection

**Figure-1:**
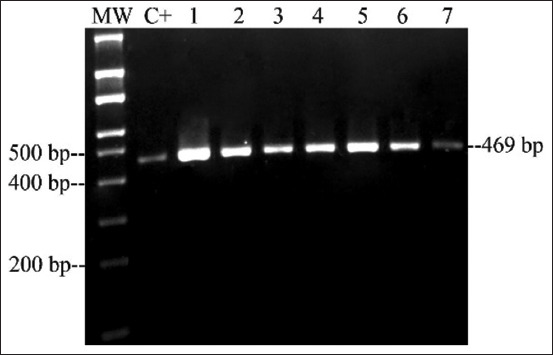
Polymerase chain reaction amplification of *qnrB* gene of seven *Salmonella* isolates. Lane C+: positive control; lane 1: *Salmonella* Heidelberg; lanes 2-7: *Salmonella* Paratyphi B; MW: 100 bp DNA Ladder (Corpogen, Colombia).

**Figure-2:**
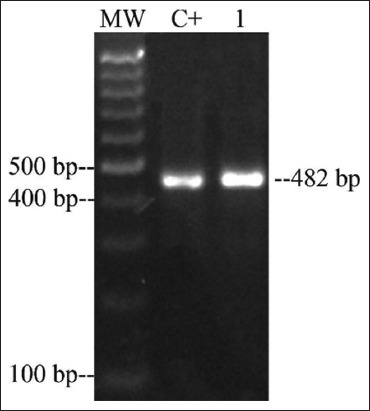
Polymerase chain reaction amplification of *aac(6’)-Ib* gene from *Salmonella* Heidelberg isolate. Lane C+: positive control; lane 1: *Salmonella* Heidelberg; MW: 100 bp DNA ladder (Solis BioDyne, Estonia).

**Figure-3:**
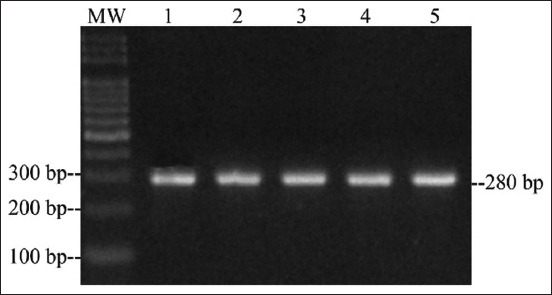
Polymerase chain reaction amplification of integrase from five *Salmonella* Heidelberg isolates. Lane 1-5: S. Heidelberg; MW: 100 bp DNA ladder (Solis BioDyne, Estonia).

The *qnrA*, *qnrC*, *qnrD*, and *qnrS* genes were not detected in any of *Salmonella* spp. isolates. In contrast, 49% of the samples were positive for the *qnrB* gene (24/49), which were distributed in two serotypes belonging to *S*. Heidelberg from broiler farms in Santander (n=3) and *S*. Paratyphi B from broiler farms in Tolima (n=21) (Table-2; Figure-1). In addition, one *S*. Heidelberg was positive for the *aac(6’)-Ib* gene (Figure-2), and the Class 1 integron was present in 11 strains isolated from Santander broiler farms (Table-2 and Figure-3).

## Discussion

Quinolones and fluoroquinolones have great importance due to their use in both human medicine and veterinary medicine all over the world. These groups of antibiotics are classified as being of the “highest priority” according to Critically Important Antimicrobials for Human Medicine published by the World Health Organization [23]. In terms of the criteria for settling on this classification, it is mentioned that quinolone and fluoroquinolone can be the sole or one of few available therapies to treat serious bacterial infections in people infected from non-human sources.

Regarding phenotypic resistance to members of the fluoroquinolone family, the rate of levofloxacin resistance was 57.1% (n= 16/28), which is higher than that reported by Donado *et al*. [24] in poultry farms from Colombia, where the antibiotic resistance levels showed values of 2.3% and 0% in the regions of Cundinamarca and Santander, respectively. The antibiotic resistance found in this study was similar to that in reports on poultry meat marketed in six Colombian cities, where the rate of resistance was 44.2% [25]. Regarding ciprofloxacin, 75% of the isolates were resistant (n=36/49), which was higher than reported in poultry farms from Brazil [26]. The ciprofloxacin resistance identified in this study agrees with the results in Colombian poultry farms from the regions of Cundinamarca (56.8%) and Santander (40.9%) [24] and in chicken carcasses marketed in Ibagué (Colombia), where the resistance was more than 42% (n=20/47) [10].

In this study, 38.8% (n=19/49) of the isolates showed resistance to enrofloxacin, which is a higher rate than reported in poultry farms in Canada (0%) [27] and Brazil, where the resistance rates ranged from 4.9% [28] to 18.2% [29]. In addition, the rate of resistance to enrofloxacin was lower than reported in two poultry-producing regions in Colombia by Donado *et al*. [24], where the resistance rates were 84.1% and 66.7% in the regions of Cundinamarca and Santander, respectively.

In the case of genotypic resistance, the *qnr* genes encode proteins of the pentapeptide repeat family, which have the ability to bind to DNA gyrase and topoisomerase IV and protect them against fluoroquinolones when the antibiotic concentration is low (0.75 µM) and Qnr protein is high (2.01 µM) [30], due to the protection is inversely proportional [31,32]. Regarding these genes, *qnrA*, *qnrB*, *qnrS*, *qnrC*, and *qnrD* have been described [9], but in our study, only the *qnrB* gene was detected in 24 out of 49 strains (49%), corresponding to *S*. Heidelberg and *S*. Paratyphi B isolated from Santander and Tolima broiler farms (Table-2). Our results showed a high rate of this gene in comparison to the results obtained in isolates from chicken products and human patients in 13 European countries, where 11.35% (n*=*138/1215) of isolates were positive for *qnrB* variants [32]. This rate is also high compared with that in strains isolated from food, animals, and humans, where the gene was present in 4.7% (n=6/129) of isolates [33]. In the case of Colombia, our results are higher than reported by Karczmarczyk *et al*. [34] in *Salmonella* isolated from commercial food, where 30% of the strains (n=4/13) belonging to *Salmonella* Infantis and *Salmonella* Uganda showed the presence of the *qnrB* gene. The high frequency of the *qnrB* gene in this study agrees with the previous studies in which this gene was the most commonly found in the *qnr* family of Enterobacteriaceae [35].

In addition, 14 strains corresponding to *S*. Heidelberg and *S*. Paratyphi B showed phenotypic resistance to fluoroquinolone antibiotics without the presence of the PMQR gene, which may indicate that the resistance could be mediated by other genes different from those assessed in this study, but resistance to this antibiotic family can also be conferred through mutations in DNA gyrase, topoisomerase IV, or efflux pumps [31]. In contrast, one of the strains of *S*. Paratyphi B was not resistant to the fluoroquinolone antibiotics evaluated in this study, but was found to possess a *qnrB* gene. Correia *et al*. [36] mentioned that this could be because the PMQR genes confer low-level quinolone resistance that alone do not allow being an effective resistance mechanism.

Aminoglycoside 6´-N-acetyltransferase [AAC(6’)-Ib] belongs to subfamily C of N-acetyltransferase (AAC), whose members are all monomeric enzymes [37]. AAC is known to catalyze the addition of an acetyl group from AcCoA to the 6´-N of aminoglycosides, disrupting the crucial electrostatic and hydrogen bonding interactions [38,39]. In our study, the *aac(6’)-Ib* gene was found only in one isolate (n=1/49) of *S*. Heidelberg from Santander broiler farms (Table-2). This prevalence is low compared with that in a report from Brazil [40], where 23 out of 129 strains showed the presence of the gene. The isolate that carried the *aac(6’)-Ib* gene was phenotypically resistant to levofloxacin and ciprofloxacin. Vetting *et al*. [41] showed that the *aac(6’)-Ib-cr* variant of this gene can confer resistance to fluoroquinolones, such as ciprofloxacin and levofloxacin, and this resistance can be related to stimulation of chromosomal mutations of the fluoroquinolones’ targets, topoisomerase IV, and DNA gyrase [42].

Class 1 integron is an important mobile element with a role in antibiotic resistance of bacteria [43]. It has been reported in *Salmonella* spp. isolates derived from poultry and other sources [44-46]. In this study, Class 1 integron was found in 11 out of the 49 strains (22.44%), which is a higher rate than reported in poultry farms in Uganda, where six out of 54 isolates showed the presence of the gene (11%) [44]; in chicken farms in Egypt, where 4.4% of *S*. Typhimurium strains were positive for this gene (n=3/67) [45]; and in Morocco, where one out of 26 isolates carried the integron (3.84%), corresponding to *S*. Infantis isolated from turkey meat [46]. These findings raise concerns about Class 1 integrons contributing to the acquisition and propagation of resistance genes in different serotypes, such as *S*. Typhimurium, *S*. Enteritidis, and *S*. Heidelberg [47].

## Conclusion

This study reports the presence of the *qnrB* gene in *S*. Heidelberg and *S*. Paratyphi B strains isolated from broiler farms in Santander and Tolima, Colombia, which may contribute to resistance to fluoroquinolones. In addition, the presence of mobile elements such as Class 1 integrons may contribute to the dissemination of resistance genes between strains.

## Authors’ Contributions

ISR and MPH: Study design. MPH: Performed the experiments, the laboratory analyses. LCF: Responsible for the phenotypic resistance experiments. ISR: Administered the project. REC, LCF, and RR: Collected the strains. ISR and MPH: Wrote the manuscript. ISR, MPH, REC, and RR: Reviewed and edited the paper. ISR: Revised the manuscript critically. All authors read and approved the final manuscript.

## References

[1] Andino A, Hanning I (2015). *Salmonella enterica*:Survival, colonization, and virulence differences among serovars. ScientificWorldJournal.

[2] Antunes P, Mourão J, Campos J, Peixe L (2016). Salmonellosis:The role of poultry meat. Clin. Microbiol. Infect.

[3] World Health Organization (2017). Non-typhoidal *Salmonella* Page.

[4] Majowicz S.E, Musto J, Scallan E, Angulo F.J, Kirk M, O'Brien S.J, Jones T.F, Fazil A, Hoekstra R.M (2010). The global burden of non-typhoidal *Salmonella* gastroenteritis. Clin. Infect. Dis.

[5] Instituto Nacional de Salud (2018). Informe de Vigilancia por Laboratorio de *Salmonella* spp. Dirección Redes en Salud Pública.

[6] Liu Y, Jiang J, Ed-Dra A, Li X, Peng X, Xia L, Guo Q, Yao G, Yue M (2021). Prevalence and genomic investigation of *Salmonella* isolates recovered from animal food-chain in Xinjiang, China. Food Res. Int.

[7] Arenas N.E, Melo V.M (2018). Producción pecuaria y emergencia de antibiótico resistencia en Colombia:Revisión sistemática. Infect.

[8] Karraouan B, Ziyate N, Ed-dra A, Amajoud N, Boutaib R, Akil A, El Allaoui A, El Ossmani H, Zerouali K, Elmdaghri N, Bouchrif B (2017). *Salmonella* Kentucky:Antimicrobial resistance and molecular analysis of clinical, animal and environment isolates, Morocco. J. Infect. Dev. Ctries.

[9] Redgrave L.S, Sutton S.B, Webber M.A, Piddock L (2014). Fluoroquinolone resistance:Mechanisms, impact on bacteria, and role in evolutionary success. Trends Microbiol.

[10] Cortés D, Rodríguez V, Verjan N (2017). *Salmonella* from chicken carcasses marketed. Rev. Bras. Cienc. Avíc.

[11] Fandiño L, Verjan N (2019). A common *Salmonella* Enteritidis sequence type from poultry and human gastroenteritis in Ibagué, Colombia. Biomédica.

[12] Rodríguez J, Rondón I, Verjan N (2015). Serotypes of *Salmonella* in broiler carcasses marketed at Ibagué, Colombia. Rev. Bras. Cienc. Avíc.

[13] Herrera-Sánchez M.P, Rodríguez-Hernández R, Rondón-Barragán I.S (2020). Molecular characterization of antimicrobial resistance and enterobacterial repetitive intergenic consensus-PCR as a molecular typing tool for *Salmonella* spp. isolated from poultry and humans. Vet. World.

[14] Lozano-Villegas K, Rodríguez-Hernández R, Rondón-Barragán I (2019). Effectiveness of six molecular typing methods as epidemiological tools for the study of *Salmonella i*solates in two Colombian regions. Vet. World.

[15] Rodríguez-Hernández R, Bernal J.F, Cifuentes J.F, Fandiño L.C, Herrera-Sánchez M.P, Rondón-Barragán I, Verjan-Garcia N (2021). Prevalence and molecular characterization of *Salmonella* isolated from broiler farms at the Tolima Region-Colombia. Animals.

[16] Castro R.E, Fandiño L.C, Vega A, Rondón I.S (2019). Phenotypic and genotypic resistance of *Salmonella* Heidelberg isolated from one of the largest poultry production region from Colombia. Int. J. Poult. Sci.

[17] Clinical and Laboratory Standards Institute (2017). Antimicrobial Susceptibility Testing (AST) Standards.

[18] Robicsek A, Strahilevitz J, Sahm D.F, Jacoby G.A, Hooper D.C (2006). qnr prevalence in ceftazidime-resistant Enterobacteriaceae isolates from the United States. Antimicrob. Agents Chemother.

[19] Kim H.B, Park C.H, Kim C.J, Kim E.C, Jacoby G.A, Hooper D.C (2009). Prevalence of plasmid-mediated quinolone resistance determinants over a 9-year period. Antimicrob. Agents Chemother.

[20] Cavaco L.M, Hasman H, Xia S, Aarestrup F.M (2009). qnrD, a novel gene conferring transferable quinolone resistance in *Salmonella enterica* serovar Kentucky and Bovismorbificans strains of human origin. Antimicrob. Agents Chemother.

[21] Park C.H, Robicsek A, Jacoby G.A, Sahm D, Hooper D.C (2006). Prevalence in the United States of aac(6')-Ib-cr encoding a ciprofloxacin-modifying enzyme. Antimicrob. Agents Chemother.

[22] Lévesque C, Roy H, Persing D, Smith T.F, Tenover F, White T (1993). PCR analysis of integrons. Diagnostic Molecular Microbiology:Principles and Applications.

[23] World Health Organization (2018). Critically Important Antimicrobials for Human Medicine.

[24] Donado P, Gardner I, Byrne B.A, Leon M, Perez E, Ovalle M.V, Tafur M.A, Miller W (2012). Prevalence, risk factors, and antimicrobial resistance profiles of *Salmonella* from commercial broiler farms in two important poultry-producing regions of Colombia. J. Food Prot.

[25] Donado P, Clavijo V, León M, Arevalo A, Castellanos R, Bernal J, Tafur M, Ovalle M.V, Alalli W, Hume M, Romero J.J, Walls I, Doyle M.P (2014). Counts, serovars, and antimicrobial resistance phenotypes of *Salmonella* on raw chicken meat at retail in Colombia. J. Food Prot.

[26] Voss-Rech D, Vaz C.L, Alves L, Coldebella A, Leão J.A, Rodrigues D.P, Back A (2015). A temporal study of *Salmonella enterica* serotypes from broiler farms in Brazil. Poult. Sci.

[27] Amand J.A, Otto S.J, Cassis R, Christianson C.B (2013). Antimicrobial resistance of *Salmonella enterica* serovar Heidelberg isolated from poultry in Alberta. Avian Pathol.

[28] Fitch F.M, Carmo-Rodrigues M.S, Oliveira V.G, Gaspari M.V, Dos Santos A, de Freitas J.B, Pignatari A.C (2016). b-lactam resistance genes:Characterization, epidemiology, and first detection of blaCTX-M-1 and blaCTX-M-14 in *Salmonella* spp. isolated from poultry in Brazil-Brazil ministry of agriculture's pathogen reduction program. Microb. Drug Resist.

[29] Biffi C, Stefani L, Miletti L, Matiello C.A, Backes R.G, Almeida J.M, Neves G.B (2014). Phenotypic and genotypic resistance profile of *Salmonella* Typhimurium to antimicrobials commonly used in poultry. Rev. Bras. Cienc. Avic.

[30] Tran J.H, Jacoby G.A (2002). Mechanism of plasmid-mediated quinolone resistance. Proc. Natl. Acad. Sci. U. S. A.

[31] Hooper D.C, Jacoby G.A (2015). Mechanisms of drug resistance:Quinolone resistance. Ann. N. Y. Acad. Sci.

[32] Veldman K, Cavaco L.M, Mevius D, Battisti A, Franco A, Botteldoorn N, Bruneau M, Perrin-Guyomard A, Cerny T, de Frutos Escobar C, Guerra B, Schroeter A, Gutierrez M, Hopkins K, Myllyniemi A, Sunde M, Wasyl D, Aarestrup F.M (2011). International collaborative study on the occurrence of plasmid-mediated quinolone resistance in *Salmonella enterica* and *Escherichia coli* isolated from animals, humans, food and the environment in 13 European countries. J. Antimicrob. Chemother.

[33] Pribul B.R, Festivo M.L, Rodrigues M.S, Costa R.G, Rodrigues E.C, de Souza M.S, Rodrigues D (2017). Characteristics of quinolone resistance in *Salmonella* spp. isolates from the food chain in Brazil. Front. Microbiol.

[34] Karczmarczyk M, Martins M, McCusker M, Mattar S, Amaral L, Leonard N, Aarestrup F.M, Fanning S (2010). Characterization of antimicrobial resistance in *Salmonella enterica* food and animal isolates from Colombia:Identification of a qnrB19-mediated quinolone resistance marker in two novel serovars. FEMS Microbiol. Lett.

[35] Cruz G.R, Radice M, Sennati S, Pallecchi L, Rossolini G.M, Gutkind G, Di Conza J.A (2013). Prevalence of plasmid-mediated quinolone resistance determinants among oxyiminocephalosporin-resistant *Enterobacteriaceae* in Argentina. Mem. Inst. Oswaldo Cruz.

[36] Correia S, Poeta P, Hébraud M, Capelo J.L, Igrejas G (2017). Mechanisms of quinolone action and resistance:where do we stand?. J. Med. Microbiol.

[37] Smith C.A, Bhattacharya M, Toth M, Stewart N.K, Vakulenko S.B (2017). Aminoglycoside resistance profile and structural architecture of the aminoglycoside acetyltransferase AAC(6')-Im. Microb. Cell.

[38] Sarno R, Hongphuc H, Weinsetel N, Tolmasky M (2003). Inhibition of aminoglycoside 6'-N-acetyltransferase type Ib-mediated amikacin resistance by antisense oligodeoxynucleotides. Antimicrob. Agents Chemother.

[39] Vong K, Auclair K (2012). Understanding and overcoming aminoglycoside resistance caused by N-6'-acetyltransferase. Medchemcomm.

[40] Pribul B.R, Festivo M.L, de Souza M.M, dos Prazeres D (2016). Characterization of quinolone resistance in *Salmonella* spp. isolates from food products and human samples in Brazil. Braz. J. Microbiol.

[41] Vetting M.W, Park C.H, Hegde S.S, Jacoby G.A, Hooper D.C, Blanchard J.S (2008). Mechanistic and structural analysis of aminoglycoside N-acetyltransferase AAC(6')-Ib and its bifunctional, fluoroquinolone-active AAC(6')-Ib-cr variant. Biochemistry.

[42] Frasson I, Cavallaro A, Bergo C, Richter S.N, Palù G (2011). Prevalence of aac(6)-Ib-cr plasmid-mediated and chromosome-encoded fluoroquinolone resistance in *Enterobacteriaceae* in Italy. Gut Pathog.

[43] Gillings M.R (2014). Integrons:Past, present, and future structure of integrons. Microbiol. Mol. Biol. Rev.

[44] Odoch T, Sekse C, L'abee-Lund T.M, Hansen H.C, Kankya C, Wasteson Y (2018). Diversity and antimicrobial resistance genotypes in non-typhoidal *Salmonella* isolates from poultry farms in Uganda. Int. J. Environ. Res. Public Health.

[45] Sharkawy H, Tahoun A, El-Gohary G.A, El-Abasy M, El-Khayat F, Gillespie T, Kitade Y, Hafez H.M, Neubauer H, El-Adawy H (2017). Epidemiological, molecular characterization and antibiotic resistance of *Salmonella enterica* serovars isolated from chicken farms in Egypt. Gut Pathog.

[46] Ed-Dra A, Karraouan B, El Allaoui A, Khayatti M, El Ossmani H, Filali F.R, ElMdaghri N, Bouchrif B (2018). Antimicrobial resistance and genetic diversity of *Salmonella* Infantis isolated from foods and human samples in Morocco. J. Glob. Antimicrob. Resist.

[47] Pérez-Moreno M.O, Centelles-Serrano M.J, Cortell-Ortolá M, Ruiz J, Llovet-Lombarte M.I, Jardí-Baiges A.M, Fort-Gallifa I (2009). Multidrug resistance related to class 1 integrons in human *Salmonella enterica* serotype Typhimurium isolates and emergence of atypical sul3-associated integrons. Int. J. Antimicrob. Agents.

